# Enhanced mPGES-1 Contributes to PD-Related Peritoneal Fibrosis via Activation of the NLRP3 Inflammasome

**DOI:** 10.3389/fmed.2021.675363

**Published:** 2021-05-18

**Authors:** Qimei Luo, Qinghua Hu, Qingkun Zheng, Lewei Gong, Lijuan Su, Baojun Ren, Yongle Ju, Zhanjun Jia, Xianrui Dou

**Affiliations:** ^1^Department of Nephrology, Shunde Hospital, Southern Medical University (The First People's Hospital of Shunde), Foshan, China; ^2^Department of Gastrointestinal Surgery, Shunde Hospital, Southern Medical University (The First People's Hospital of Shunde), Foshan, China; ^3^Nanjing Key Laboratory of Pediatrics, Children's Hospital of Nanjing Medical University, Nanjing, China

**Keywords:** mPGES-1, PGE2, peritoneal fibrosis, inflammation, NLRP3 inflammasome

## Abstract

**Background:** Microsomal prostaglandin E synthase-1 (mPGES-1)-derived prostaglandin E_2_ (PGE2) is a chief mediator of inflammation. However, the role and mechanism of mPGES-1 in peritoneal dialysis (PD)-associated peritoneal fibrosis have not been investigated.

**Material and Methods:** In PD patients, mPGES-1 expression in peritoneum tissues and the levels of PGE2, IL-1β, and IL-18 in the dialysate were examined. In rat peritoneal mesothelial cells (RPMCs), the regulation and function of mPGES-1 and NLRP3 inflammasome were investigated. The expression of extracellular matrix proteins and the components of NLRP3 inflammasome were detected by Western blotting or real-time quantitative PCR.

**Results:** In PD patients with ultrafiltration failure (UFF), mPGES-1 was enhanced in the peritoneum, which was associated with the degree of peritoneal fibrosis. Accordingly, the intraperitoneal PGE2 levels were also positively related to the PD duration, serum C-reactive protein levels, and serum creatinine levels in incident PD patients. In RPMCs, high-glucose treatment significantly induced mPGES-1 expression and PGE2 secretion without affecting the expressions of mPGES-2 and cPGES. Inhibition of mPGES-1 via short hairpin RNA significantly ameliorated the expression of extracellular matrix proteins of RPMCs induced by high glucose. Additionally, high glucose markedly activated NLRP3 inflammasome in RPMCs that was blunted by mPGES-1 inhibition. Furthermore, silencing NLRP3 with siRNA significantly abrogated the expression of extracellular matrix proteins in RPMCs treated with high glucose. Finally, we observed increased IL-1β and IL-18 levels in the dialysate of incident PD patients, showing a positive correlation with PGE2.

**Conclusion:** These data demonstrate that mPGES-1-derived PGE2 plays a critical role in PD-associated peritoneal fibrosis through activation of the NLRP3 inflammasome. Targeting mPGES-1 may offer a novel strategy to treat peritoneal fibrosis during PD.

## Introduction

Peritoneal dialysis (PD) has become a well-established treatment option for patients with end-stage renal disease due to the advantages of its minimal requirements for technical support and better preservation of residual renal function (1). However, long-term PD is limited because chronic exposure to bioincompatible PD fluid can lead to obvious peritoneal fibrosis (2). These changes in the peritoneum include the loss of the mesothelial monolayer, thickening of the submesothelial layer because of increased deposition of the extracellular matrix, and angiogenesis (3–5). Fibrosis of the peritoneal membrane can culminate in peritoneum ultrafiltration failure (UFF), eventually forcing the withdrawal of PD therapy (6).

Accumulating evidence has implicated that sterile PD solutions containing high glucose can induce chronic sterile inflammation in the submesothelial zone, leading to peritoneal fibrosis (7, 8). High levels of inflammatory cytokines in the peritoneal cavity seem to be associated with the progress of peritoneal fibrosis (9). The fibrotic progress develops following chronic sterile inflammation in the peritoneum. As the first protection against biochemical and microorganism attack, peritoneal mesothelial cells incur various injuries and secrete inflammatory cytokines or chemokines, leading to the synthesis of extracellular matrix proteins and progression to peritoneal fibrosis (10, 11). Sustained peritoneal chronic inflammation is considered a potential mechanism of peritoneal fibrosis (12). Targeting regulation of peritoneal chronic inflammation may be an effective anti-fibrosis therapy in PD.

Prostaglandin E_2_ (PGE2) is an abundant prostanoid that is considered the chief mediator of inflammation. The biosynthesis of PGE2 is catalyzed by prostaglandin E synthases, that contain microsomal prostaglandin E synthase-1 (mPGES-1), microsomal prostaglandin E synthase-2 (mPGES-2) and cytosolic prostaglandin E synthase (cPGES). mPGES-2 and cPGES are constitutively expressed, whereas mPGES-1, which is the dominant synthetic enzyme for PGE2 production, is an inducible enzyme under many pathological conditions (13). mPGES-1 is involved in inflammation, pain, angiogenesis, and tumorigenesis (13–15). Evidence has shown that mPGES-1 is a critical mediator of chronic inflammation in animal models of arthritis (16). High glucose increases PGE2 synthesis in human peritoneal mesothelial cells (17), while the role of mPGES-1-derived PGE2 in high-glucose-induced peritoneal mesothelial cell injury remains to be defined.

Recently, the NOD-like receptor family, pyrin domain-containing 3 (NLRP3) inflammasome was found to be activated in the peritoneal tissues of acute bacterial peritonitis in patients on PD, and blockade of NLRP3 rescued morphologic alterations during acute peritonitis (18). The NLRP3 inflammasome plays a critical role in the acute inflammation response of the peritoneum, but the role of the NLRP3 inflammasome in the condition of chronic inflammation in the peritoneum still needs to be explored. More interestingly, PGE2 activates NLRP3 signaling in peritoneal macrophages (19). However, it remains unknown whether mPGES-1/PGE2 mediates activation of the NLRP3 inflammasome under the condition of chronic inflammation induced by PD fluid.

In the present study, we examined the expression of mPGES-1 in the peritoneum and the secretion of PGE2 in PD fluid and analyzed the correlation between the activation of mPGES-1/PGE2 and peritoneal fibrosis. Additionally, the effect of mPGES-1/PGE2 on the regulation of the NLRP3 inflammasome and cascade in high-glucose-induced fibrosis was analyzed.

## Materials and Methods

### Human Sample Collection

Peritoneum tissues from inguinal hernia patients with normal renal function, ESRD patients, and PD patients with UFF were collected at Shunde Hospital of Southern Medical University. Enrollment included patients aged 14 years and older who were diagnosed with hernia repair and needed to accept surgery therapy or ESRD patients or patients who had withdrawn from PD therapy due to UFF. Patients were excluded if they refused to give written consent or had malignant disease or had diabetes mellitus or had heterotopia endometriosis or took non-steroidal anti-inflammatory drugs (NSAIDs) in the past 2 weeks or took COX-2-selective inhibitors (e.g., celecoxib, nimesulide, meloxicam, and rofecoxib) in the past 2 weeks. Ultrafiltration failure was defined as, after a 4-h residence period with 4.25% PD fluid, the net ultrafiltration failure to achieve at least 400 mL (20). Peritoneal biopsies were collected at the time of laparoscopic inguinal hernia repair or laparoscopic for PD catheter implantation or catheter withdrawal. Peritoneal tissues of inguinal hernia patients with normal renal function were normal control samples. We also collected PD fluid samples and serum samples from 116 incident PD patients. The daily ultrafiltration volume and daily urine volume were recorded, and the ultrafiltration function of the peritoneal membrane was evaluated by carrying out 4-h PET. The nocturnal peritoneal dialysate samples and serum samples were collected on the day of PET. The peritoneal dialysate was drained after at least 8 h of dwell exchange. Serum C-reactive protein, serum hemoglobin, serum creatinine, serum calcium, dialysate creatinine, and dialysate urea nitrogen levels were assessed using an automatic biochemical instrument (AU5800; Beckman Coulter). All the patients provided informed consent. The use of human biopsy samples, serum and dialysate samples was approved by the Ethical Review Board of Shunde Hospital of Southern Medical University.

### Histology and Immunohistochemistry

Changes in the peritoneal tissues were assessed using paraformaldehyde-fixed, paraffin-embedded sections (4–μm) with hematoxylin-eosin staining (HE) or Masson's trichrome staining (Masson). The thickness of the peritoneum was determined under a microscope and was expressed as the mean of five independent measurements for each section. Immunohistochemistry was examined in paraffin sections using a microwave-based antigen retrieval technique (21). Briefly, sections (4–μm) were deparaffinized, rehydrated and incubated in 3% H_2_O_2_ for 15 min to block endogenous peroxidase. Antigen retrieval for mPGES-1 from the sections was performed using an antigen retrieval solution (1 mmol/L Tris-HCL, 0.1 mmol/L EDTA, pH 8.0) for 15 min at high power in a microwave oven. Then, sections were incubated with 5% normal goat serum (abs933, Absin, China) in PBS for 60 min. Subsequently, sections were incubated overnight at 4°C with a rabbit anti- mPGES-1 antibody (no. ab203247, Abcam, England) or a isotype IgG (no. 3900s, Cell Signaling Technology), followed by the incubation with secondary antibody at room temperature for 1 h, and the signal was visualized using a DAB kit (no. G1215-1, Servicebio, China). The optical density of mPGES-1 in the peritoneum was quantified using Image-Pro plus software. Five consecutive fields were assessed for each section, and a mean value from five independent measurements was used for statistical analysis.

### ELISA

The PGE2, IL-1β, and IL-18 protein levels were quantified using commercial kits (no. ml024761-C, ml050857-C, ml058055-C, respectively; Shanghai Enzyme-linked Biotechnology, China) following the manufacturer's instructions. PGE2 was measured in 48-h conditioned medium.

### Cell Culture

The immortalized rat peritoneal mesothelial cell (RPMC) line was from Zongpei Jiang (The Sixth Affiliated Hospital, Sun Yat-sen University, Guangzhou, China). Cells were cultured in DMEM/F12 medium containing 10% fetal bovine serum (FBS; Gibco, USA) and 1% antibiotics (100 μg/mL of streptomycin and 100 U/mL of penicillin) (Life Technologies, USA) at 37°C with 5% CO_2_. Cells were deprived of serum for 24 h and were stimulated with D-glucose (Life Technologies, USA) at a normal (5.5 mmol/L) or high (138 mmol/L) concentration for 0, 24, 48, and 72 h.

### Transient Transfection

RPMCs were seeded in a six-well plate at 70%−80% confluence before transfection. The cells were transfected with mPGES-1 shRNA (GeneCopoei, Guangzhou, China) or NLRP3 siRNA (RiboBio, Guangzhou, China) or a negative control of shRNA or siRNA using Lipofectamine 3000 (Life Technologies, USA) according to the manufacturer's instructions. In brief, for mPGES-1 shRNA plasmid transfection, the cells were transfected with 2 ug mPGES-1 shRNA plasmid or control plasmid 8 h before high glucose treatment. For NLRP3 siRNA transfection, the cells were incubated with 500 nM NLRP3 siRNA or scrambled siRNA, then the media were replaced with D-glucose at a high concentration 6 h after transfection. Next, the cells were harvested for mRNA or protein analysis at 24 h or 48 h after high glucose stimulation.

### RNA Extraction and Quantitative Real-Time PCR

Total RNA was extracted from the cultured cells with Trizol reagent (Invitrogen, USA). cDNA was synthesized from 1 μg of total RNA using the PrimeScript^TM^ RT reagent Kit (no. RR470A; TaKaRa, Japan) according to the manufacturer's protocol. Oligonucleotides were designed and synthesized by Sangon Biotech Company. Real-time PCR was performed using the SYBR Green PCR Kit (no. RR820A; TaKaRa, Japan). The sequences of the primer pairs are shown in Table 1. Amplification was performed using the CFX96 Real-Time PCR detection system (Bio-Rad, USA). The cycling conditions were as follows: 95°C for 30 s, followed by 40 cycles of 95°C for 5 s and 60°C for 30 s. The mRNA levels were normalized to β-actin as a control. The data were presented as the relative fold change compared with the control.

**Table 1:** Primer sequences for q-PCR.

**Gene symbol**	**Primer sequences**
β-actin	5′-TGTGACGTTGACATCCGTAAAG-3′
	5′-GGCAGTAATCTCCTTCTGCATC-3′
mPGES-1	5′-AAGCCTGGCCATCTGTATTT-3′
	5′-TGAGCCAGATTGTACCACTTC-3′
mPGES-2	5′-GAGAAAGCTCGCAACAACTAAAT-3′
	5′-TCATGGCTGGGTAGTAGGT-3′
cPGES	5′-CTTGTCAGTGTTCCAGGTGTAT-3′
	5′-TTTCTTCGTCCTCCATGCTAAG-3′
IL-18	5′-GAATCCCAGACCAGACTGATAAT-3′
	5′-GGTAGACATCCTTCCATCCTTC-3′
NLRP3	5′-GGAAGATGTGGACCTCAAGAAA-3′
	5′-GATCCAAGTGATCTGCCTTCTC-3′
Caspase-1	5′-AAGACAAGCCCAAGGTTATCA-3′
	5′-AAGAATCCCTCTTCGGAGTTTC-3′

### Western Blotting

The cells were washed with phosphate-buffered saline and lysed with lysis buffer (Cell Signaling Technology, USA). The protein samples were separated by SDS-PAGE and transferred to PVDF membranes (Bio-Rad). The membranes were blocked with 5% non-fat milk and then were incubated overnight with primary antibodies against mPGES-1 (no. 160140; 1:500; Cayman, USA), fibronectin (no. ab45688; 1:1000; abCam, England), Collagen I (no. ab138492; 1:500; abCam), E-cadherin (no. 14472; 1:1000; Cell Signaling Technology), NLRP3 (no. ab263899; 1:1000; abCam), procaspase-1 and caspase-1 (no. ab179515; 1:500; abCam), and β-actin (no. 3700; 1:5000; Cell Signaling Technology) in TBS with 0.1% Tween 20. Next, the membranes were incubated with horseradish peroxidase (HRP)-conjugated anti-mouse IgG (no. 7076; 1:5000; Cell Signaling Technology) or anti-rabbit IgG antibodies (no. 7074; 1:5000; Cell Signaling Technology) for 1 h at room temperature. ECL chemiluminescence (Bio-Rad) was used to visualize the proteins using the ChemiDoc XRS + system (Bio-Rad).

### Statistical Analysis

All the data were shown as mean ± SEM. Statistical analysis was performed using SPSS 20.0 for Mac. Statistical differences were assessed by ANOVA followed by the LSD test for multiple groups. The correlations mentioned in this article were assessed by the Spearman's rank correlation coefficient. *P* < 0.05 was deemed to indicate statistical significance.

## Results

### Enhanced mPGES-1 Expression in the Peritoneal Tissue of Ultrafiltration Failure PD Patients

**Figure 1:**
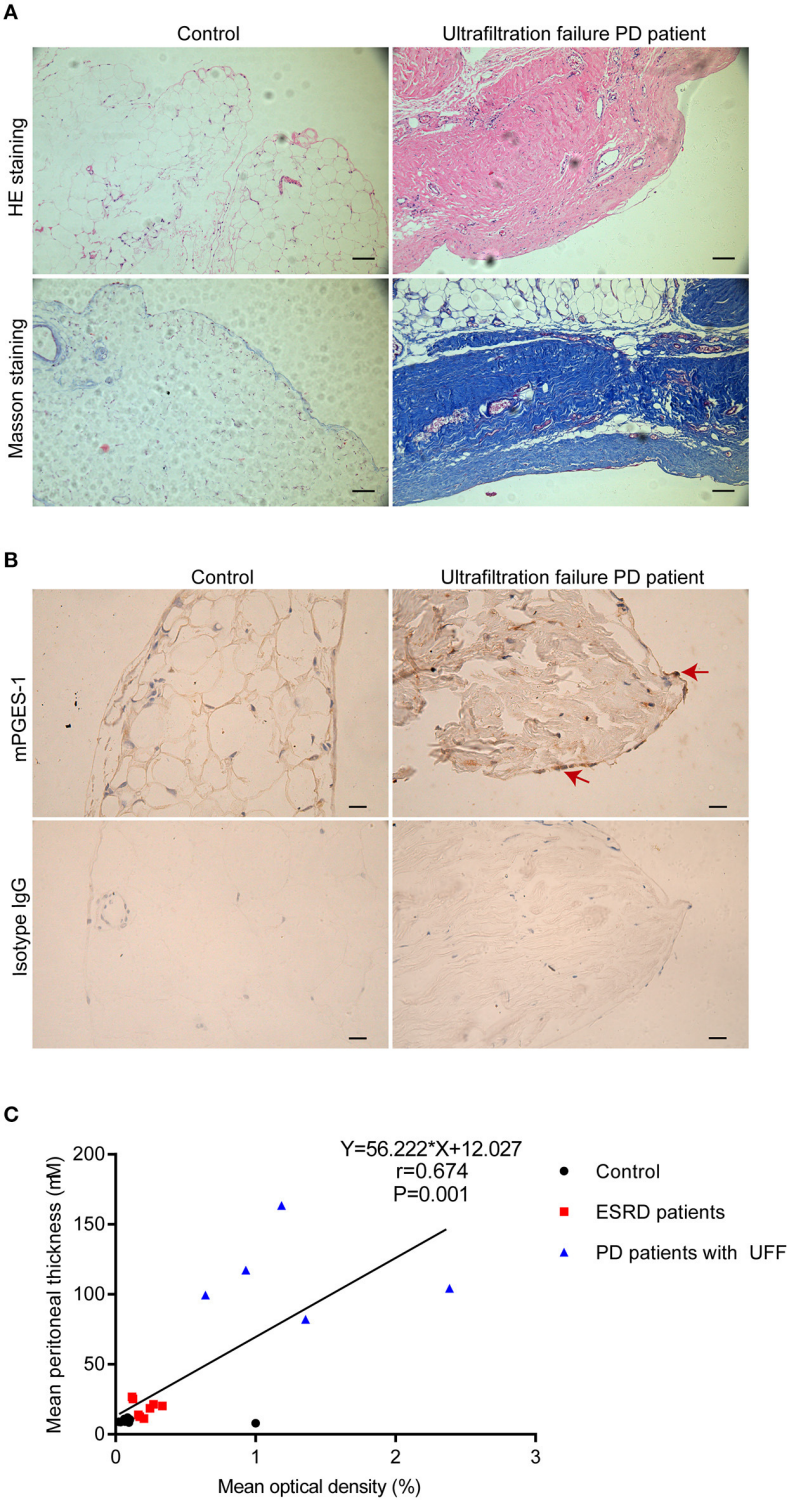
Enhanced expression of mPGES-1 in the peritoneal tissues of PD patients with ultrafiltration failure. **(A)** Representative HE and Masson staining in peritoneal tissues of inguinal hernia patients with normal renal function and PD patients with UFF. Magnification: 100×. Scale bar: 100 μm. **(B)** Representative image of immunohistochemical staining of mPGES-1 in the peritoneal tissues of inguinal hernia patients with normal renal function and PD patient with UFF. Analysis of mPGES-1 antibody specificity by replacing mPGES-1 antibody with isotype IgG. Magnification: 400×. Scale bar: 20μm. **(C)** Correlation between the expression of mPGES-1 in peritoneal tissues and the thickness of the peritoneum (Spearman's rank correlation analysis, *n* = 21). Symbols of black dot, red square, and blue triangle represented control, ESRD patients, and PD patients with UFF, respectively.

We initially studied the expression of mPGES-1 in the peritoneal tissue of inguinal hernia patients with normal renal function (control group) and PD patients with UFF. Loss of mesothelial cells and thickening of the submesothelial layer are important features of peritoneal fibrosis. HE staining and Masson's staining revealed that, compared with the peritoneal tissue of the control group, loss of the mesothelial cell monolayer and the thickness of the peritoneum significantly increased in PD patients with UFF (Figure 1A). Immunohistochemical analysis of the visceral peritoneum showed that mPGES-1 was expressed in residual mesothelial cells in ultrafiltration failure PD patients, while mPGES-1 was rarely expressed in inguinal hernia patients (Figure 1B). Furthermore, we analyzed the correlation between the expression of mPGES-1 in peritoneal tissue and thickness of the peritoneum. We collected 21 peritoneum samples from eight inguinal hernia patients, 8 ESRD patients and 5 PD patients with UFF. As shown in Figure 1C, the expression of mPGES-1 in the peritoneum and thickness of the peritoneum showed a significant positive correlation. Data about the expression of mPGES-1 and thickness of peritoneum in individual patient were shown in Supplementary Material. These results indicate that the expression of mPGES-1 may be associated with peritoneal fibrosis.

### Increased PGE2 Secretion During PD Therapy

**Figure 2:**
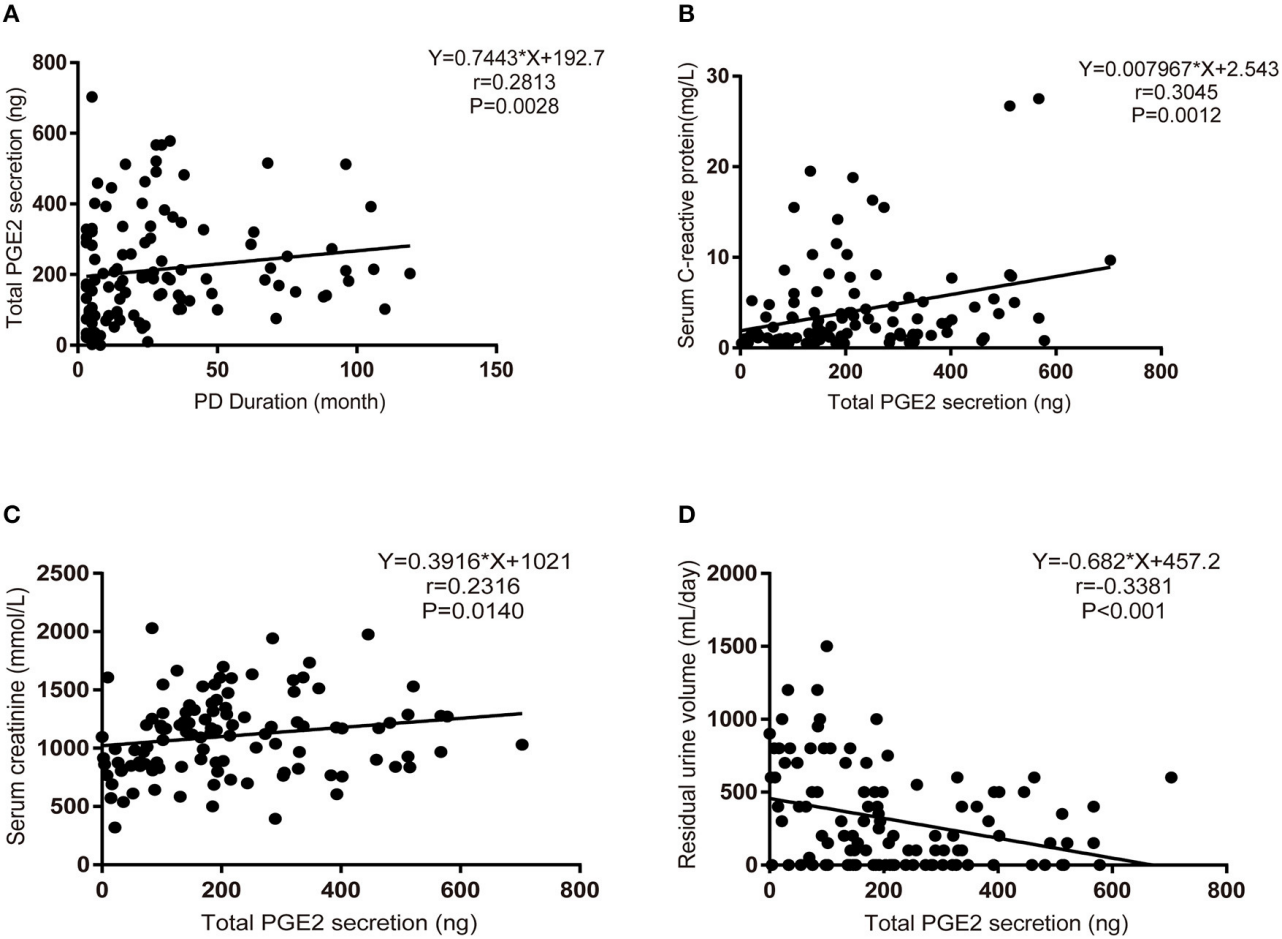
Increased PGE2 secretion during PD therapy. **(A)** Bivariate correlation analysis between the total secretion of PGE2 and the PD duration in incident PD patients (Spearman's rank correlation analysis, *n* = 116). **(B)** Bivariate correlation analysis between the total secretion of PGE2 and serum C-reactive protein levels in incident PD patients (Spearman's rank correlation analysis, *n* = 116). **(C)** Bivariate correlation analysis between the total secretion of PGE2 and serum creatinine levels in incident PD patients (Spearman's rank correlation analysis, *n* = 116). **(D)** Bivariate correlation analysis between the total secretion of PGE2 and daily residual urine volume levels in incident PD patients (Spearman's rank correlation analysis, *n* = 116).

PGE2 is the only downstream effector of mPGES-1. Next, we detected the secretion of PGE2 in PD fluid and investigated the relationship between PGE2 secretion and the clinical characteristics of PD patients. In total, 116 PD patients were enrolled [mean age, 50.65 ± 14.16 (SD) years], with a median dialysis duration of 50 (maximum, 119) months. The mean PGE2 value was 256 (range, 10.00–472.10) pg/mL, and the mean ultrafiltration volume was 800 (range, 0–2,200) mL/day for all patients (Table 2). Considering that the concentration of PGE2 was affected by the patients' ultrafiltration volume, we analyzed the total secretion of PGE2 instead of the PGE2 concentration. Bivariate correlation analysis revealed that the total secretion of PGE2 was positively correlated with the PD duration, serum C-reactive protein level, and serum creatinine level but negatively correlated with the patients' daily residual urine volume (Figures 2A–D). These data demonstrate that the total secretion of PGE2 in PD fluid increases during PD therapy.

**Table 2:** Patients' clinical characteristics.

	***N* = 116**
Age (year)	50.65 ± 14.16
Male (%)	66 (56.8%)
BMI (kg/m^2^)	21.80 (19.90, 24.20)
Dialysis duration (month)	50.00 (39.25, 61.75)
Dialysate PGE2 (pg/mL)	256.18 ± 101.95
Ultrafiltration volume (mL/day)	800.00 (432.50, 1096.25)
Mean arterial pressure (mmHg)	99.67 (92.33, 105.50)
Serum C-reactive protein (mg/L)	2.34 (0.99, 5.08)
Serum hemoglobin (g/L)	110.00 (95.50, 120.00)
Serum creatinine (umol/L)	1089.02 ± 343.35
Albumin-corrected calcium (mmol/L)	2.30 (2.15, 2.46)
Total Kt/V	1.98 (1.72, 2.35)
Residual urine volume (mL/day)	200.00 (0.00, 537.50)

### Induction of mPGES-1 Expression by High Glucose in RPMCs

**Figure 3:**
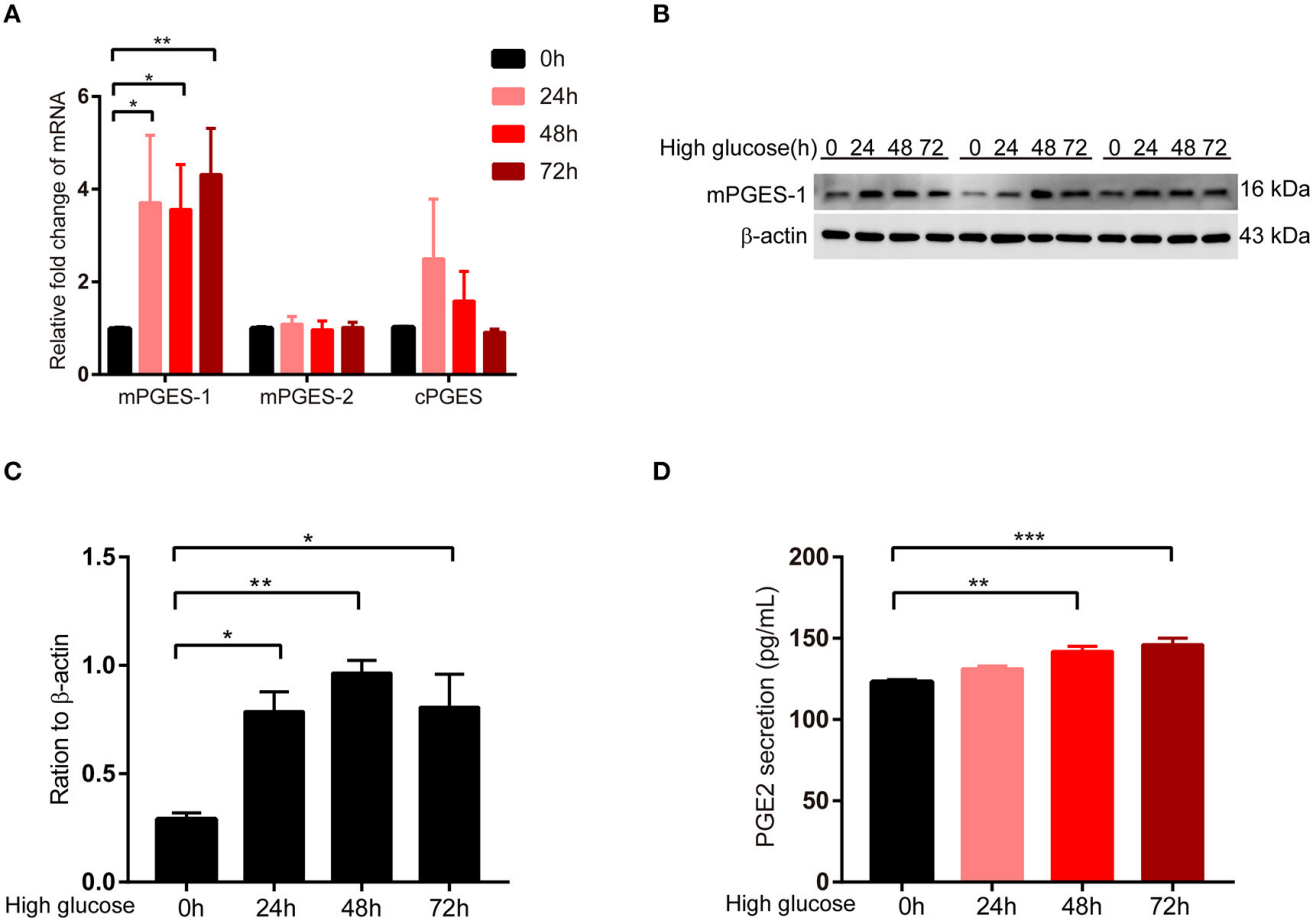
Induction of mPGES-1 expression by high glucose in RPMCs. **(A)** Real-time quantitative PCR analysis of mPGES-1, mPGES-2, and cPGES levels in RPMCs in response to high glucose (138 mmol/L) for the indicated times. **(B)** RPMCs were incubated with high glucose (138 mmol/L) for the indicated times, and western blotting detected the expression of mPGES-1. **(C)** Quantitative analysis of mPGES-1 expression in RPMCs treated with high glucose (138 mmol/L) for the indicated times. **(D)** RPMCs were treated with high glucose (138 mmol/L) for the indicated times, and ELISA was performed for PGE2 in the cell culture medium. The data are represented as the mean ± SEM. *P < 0.05, **P < 0.01, and ***P < 0.001.

Next, we detected the expression change of mPGES-1 in RPMCs after high glucose damage. First, the expression levels of mPGES-1, mPGES-2, and cPGES in RPMCs under high glucose were detected. After treating RPMCs with glucose at the concentration of 138 mmol/L for 0, 24, 48, and 72 h, the mRNA expression of mPGES-1, but not those of mPGES-2 and cPGES, was significantly up-regulated as determined by quantitative RT–PCR (Figure 3A). Western blotting showed that the expression level of mPGES-1 was significantly increased in high-glucose-induced RPMCs (Figures 3B,C), and ELISA showed that the secretion of PGE2 in supernatants was markedly elevated in a time-dependent manner in RPMCs treated with high glucose (Figure 3D).

### Inhibition of mPGES-1 Ameliorates High-Glucose-Induced Synthesis of Extracellular Matrix Proteins in RPMCs

**Figure 4:**
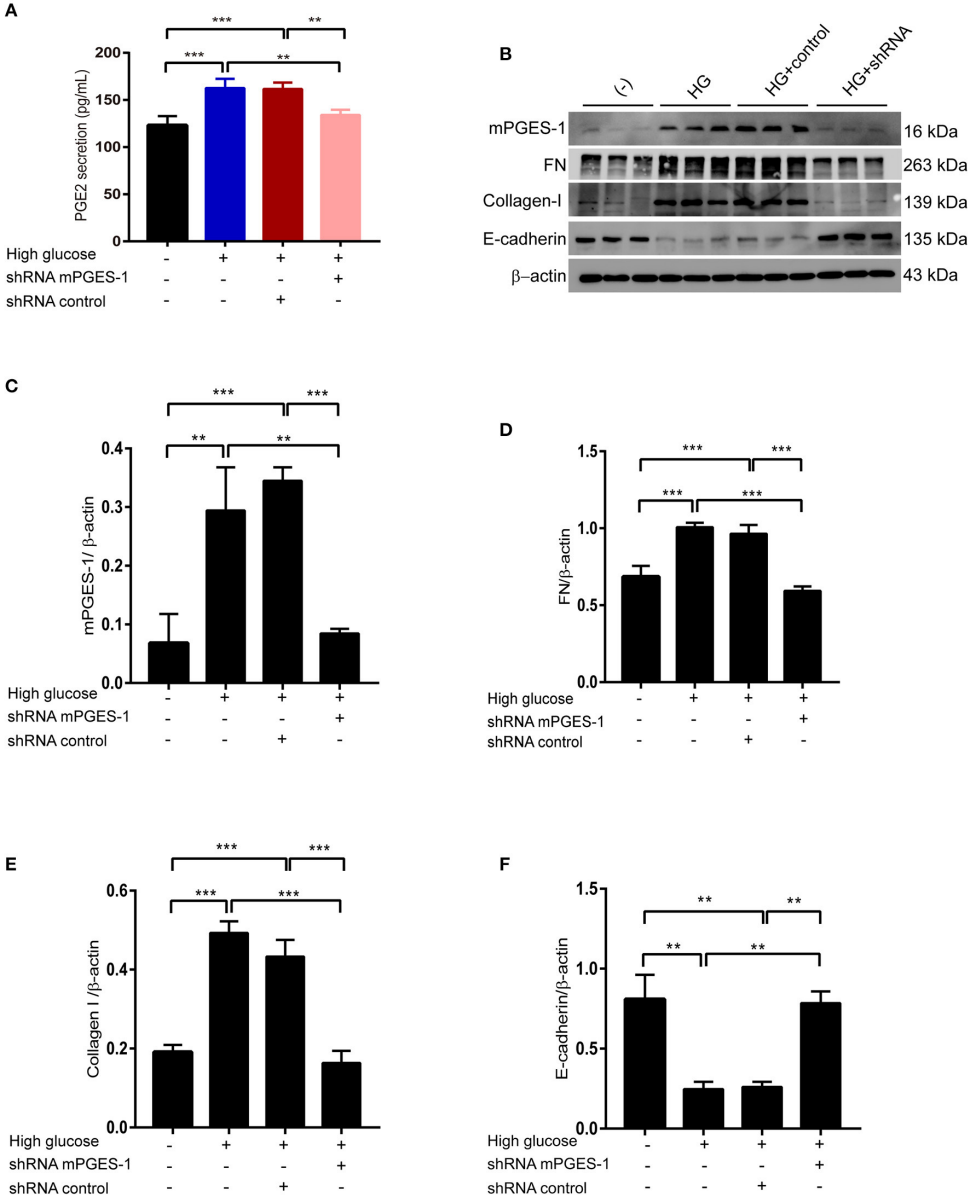
Inhibition of mPGES-1 ameliorates high-glucose-induced synthesis of extracellular matrix proteins in RPMCs. **(A)** RPMCs were treated with mPGES-1 shRNA plasmid or control shRNA plasmid, followed by incubation with high glucose for 48 h. ELISA of PGE2 in cell culture medium in RPMCs treated with normal glucose, high glucose, high glucose plus shRNA control or high glucose plus mPGES-1 shRNA. **(B)** RPMCs were transfected with the mPGES-1 shRNA plasmid or control shRNA plasmid, followed by incubation with high glucose for 48 h. Western blotting confirmed the expression of mPGES-1, FN, Collagen-I, and E-cadherin. Quantitative analyses of mPGES-1, FN, Collagen-I, and E-cadherin were displayed in the **(C–F)**. The data are represented as the mean ± SEM. **P < 0.01, ***P < 0.001.

To further analyze the contribution of mPGES-1/PGE2 signaling to peritoneal fibrosis, we analyzed its effect on the synthesis of extracellular matrix proteins in RPMCs after transfection with mPGES-1 shRNA or control shRNA. The PGE2 release in supernatants was significantly elevated in response to high-glucose treatment (Figure 4A). This increase in PGE2 was abolished by silencing mPGES-1 (Figure 4A). As shown in Figures 4B,C, mPGES-1 shRNA significantly reduced the expression of mPGES-1 in the high-glucose condition. By application of mPGES-1 shRNA, high-glucose-induced upregulation of fibronectin (FN) and collagen I was significantly blunted (Figures 4B,D,E), while high-glucose-induced downregulation of E-cadherin was significantly increased (Figures 4B,F).

### Inhibition of mPGES-1 Blunts High-Glucose-Induced Activation of the NLRP3 Inflammasome

**Figure 5:**
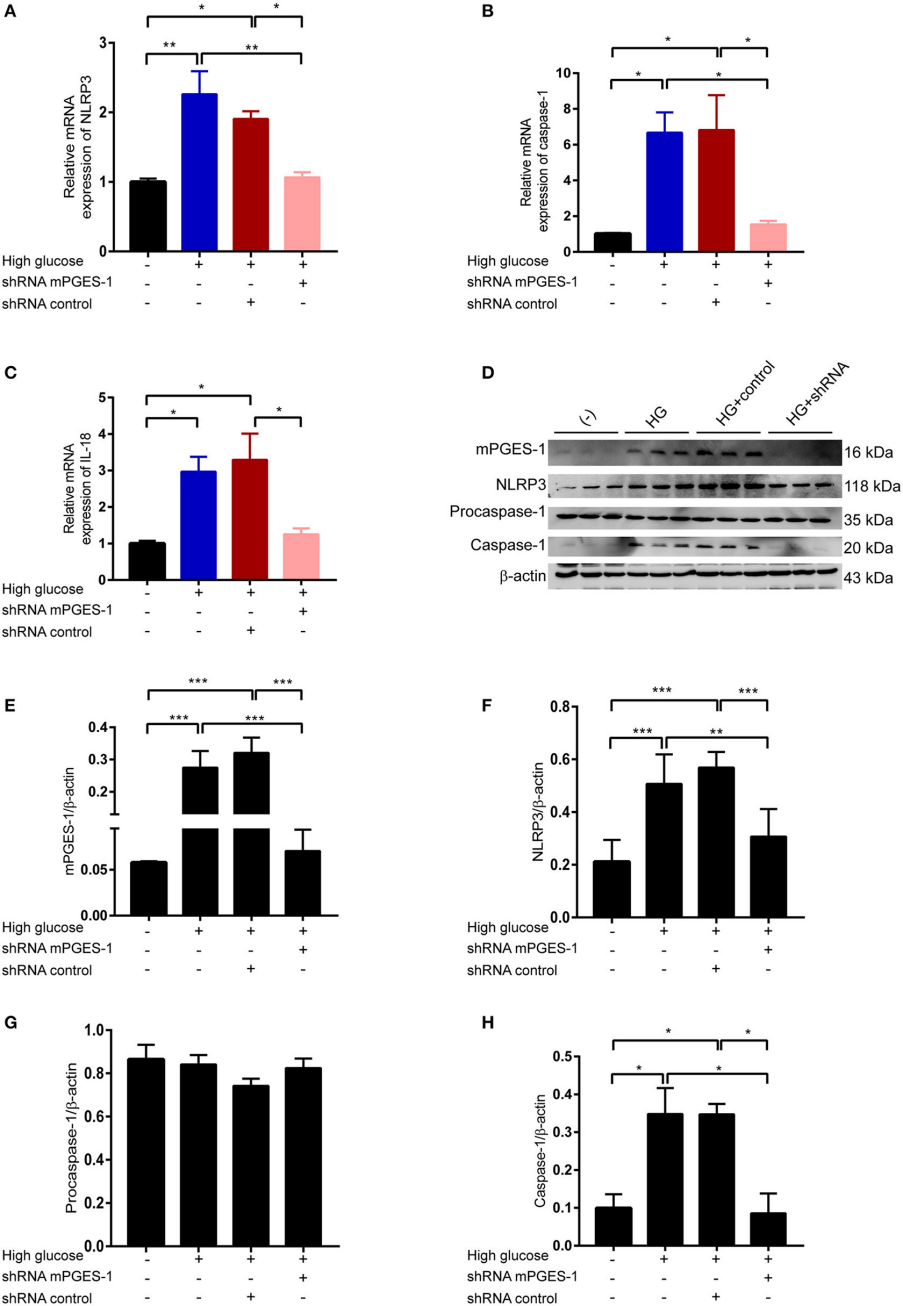
Inhibition of mPGES-1 blunts high-glucose-induced activation of the NLRP3 inflammasome. **(A–C)** RPMCs were transfected with the mPGES-1 shRNA plasmid or control shRNA plasmid, followed by incubation with high glucose for 24 h. Real-time quantitative PCR analysis was used to detect the mRNA levels of NLRP3 **(A)**, caspase-1 **(B)**, and IL-18 **(C)**. **(D–H)** RPMCs were transfected with the mPGES-1 shRNA plasmid or control shRNA plasmid, followed by incubation with high glucose for 48 h. Western blotting confirmed the expression of mPGES-1, NLRP3, procaspase-1, and caspase-1. Quantitative analyses of mPGES-1 **(E)**, NLRP3 **(F)**, procaspase-1 **(G)**, and caspase-1 **(H)** expression were displayed. The data are represented as the mean ± SEM. *P < 0.05, **P < 0.01, ***P < 0.001.

We next investigated how mPGES-1 mediates extracellular matrix protein synthesis in RPMCs. High-glucose-upregulated mRNA levels of NLRP3, activated caspase-1, and IL-18 were significantly blunted in RPMCs transfected with mPGES-1 shRNA (Figures 5A–C). After treating RPMCs with high glucose, the expression of NLRP3 was induced (Figure 5D). Western blotting further confirmed the induction of NLRP3 and activated caspase-1 at the protein levels, a phenomenon that was remarkably reduced by mPGES-1 shRNA (Figures 5D–H). There was no significantly change of procaspase-1 protein in RPMCs treated with mPGES-1 shRNA.

### Inhibition of NLRP3 by siRNA Abrogates High-Glucose-Induced Synthesis of Extracellular Matrix Proteins in RPMCs

**Figure 6:**
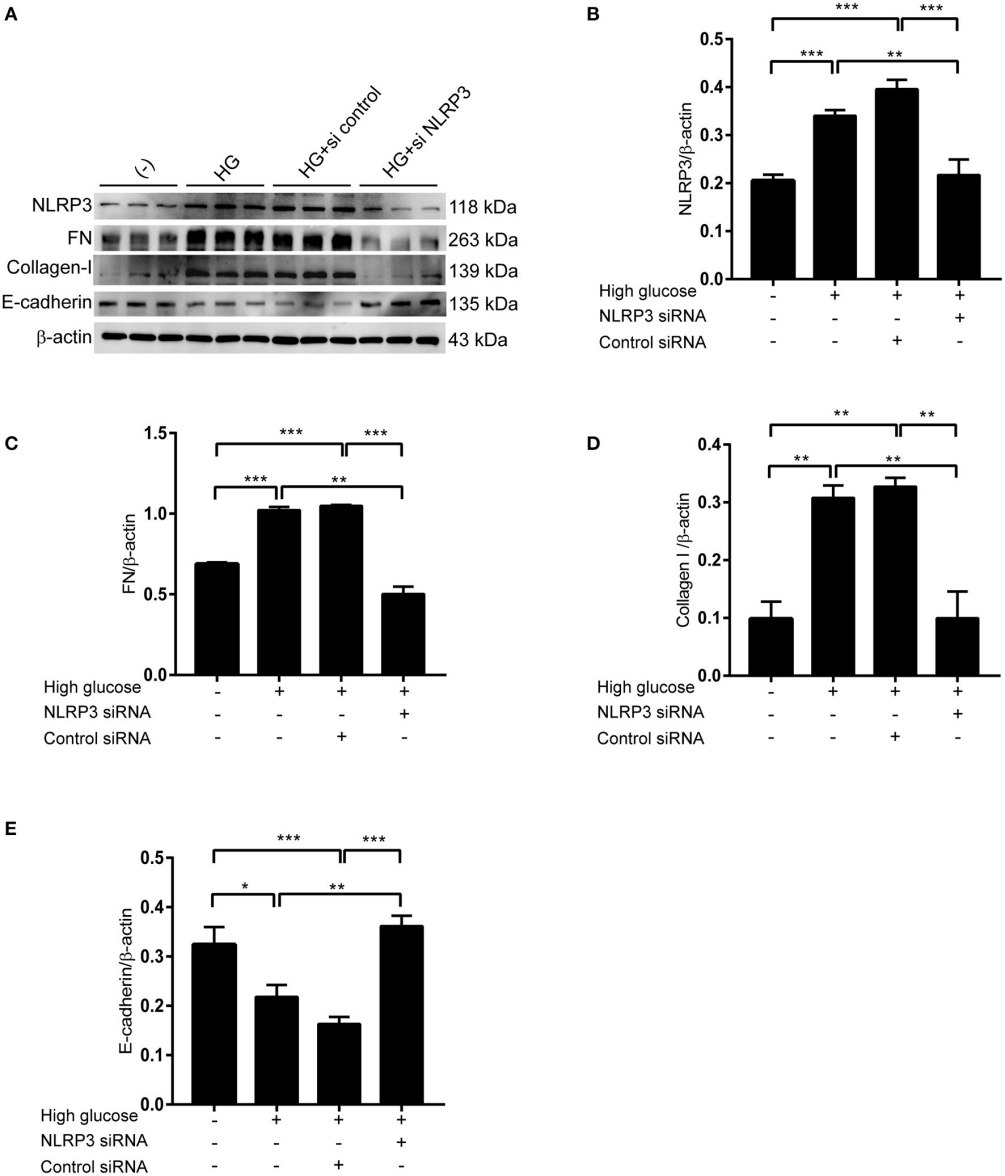
Inhibition of NLRP3 by siRNA abrogates high-glucose-induced synthesis of extracellular matrix proteins in RBMCs. **(A)** RBMCs were treated with NLRP3 siRNA or control siRNA, followed by incubation with high glucose for 48 h. Western blotting confirmed the expression of NLRP3, FN, Collagen-I, and E-cadherin. Quantitative analyses of NLRP3, FN, Collagen-I, and E-cadherin were displayed in the **(B–E)**. The data are represented as the mean ± SEM. *P < 0.05, **P < 0.01, ***P < 0.001.

Our data suggest that mPGES-1 can mediate the activation of the NLRP3 inflammasome. We next explored whether the NLRP3 inflammasome is involved in high-glucose-induced synthesis of extracellular matrix proteins in RPMCs. NLRP3 siRNA was applied to inhibit the expression of NLRP3 in RPMCs treated with high glucose. Western blotting confirmed the induction of FN and collagen-I protein levels under high glucose was significantly reduced by NLRP3 siRNA treatment, and the reduction of E-cadherin protein levels under high glucose was significantly reversed (Figures 6A–E).

### Positive Correlation Between the Secretion of PGE2 and IL-1β or IL-18 in PD Fluid

**Figure 7:**
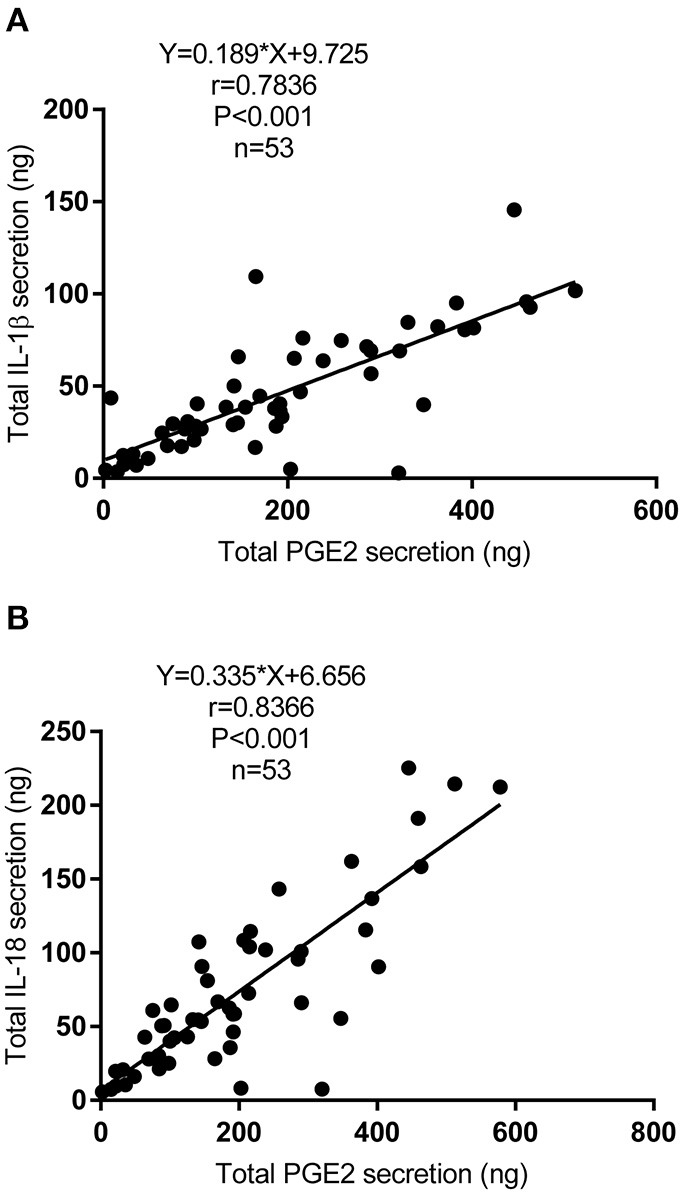
Correlation between the secretion of PGE2 and IL-1β or IL-18 in PD fluid. **(A)** Bivariate correlation analysis between the total secretion of PGE2 and IL-1β in incident PD patients' dialysate (Spearman's rank correlation analysis, *n* = 53). **(B)** Bivariate correlation analysis between the total secretion of PGE2 and IL-18 in incident PD patients' dialysate (Spearman's rank correlation analysis, *n* = 53).

IL-1β and IL-18 are the major products of the activation of the NLRP3 inflammasome. We next detected the secretion of IL-1β and IL-18 in the dialysate and explored the relationship between the secretion of PGE2 and IL-1β or IL-18 in PD fluid. Fifty-three patients were enrolled [mean age, 46.43 ± 13.30 (SD) years], and their mean concentrations of IL-1β and IL-18 were 69.12 and 107.48 pg/L, respectively. IL-1β and IL-18 were corrected by ultrafiltration volumes because of the difference in the daily ultrafiltration volume in PD patients. As shown in Figure 7, the total secretion of PGE2 was positively correlated with the total secretion of IL-1β (*r* = 0.7836; *P* < 0.001) (Figure 7A) and total secretion of IL-18 (*r* = 0.8366; *P* < 0.001) (Figure 7B) in the dialysate.

## Discussion

In this study, we combined clinical and functional data in humans and RPMCs to demonstrate that mPGES-1/PGE2 signaling is activated in PD patients with UFF and the expression of mPGES-1 in the peritoneum is positively correlated with peritoneal fibrosis. Inhibition of mPGES-1 *in vitro* ameliorated high-glucose-induced synthesis of extracellular matrix proteins in RPMCs through blunting the activation of the NLRP3 inflammasome. Compared with traditional NSAIDs, targeting mPGES-1 may be a safer and more effective alternative to control acute inflammation, and mPGES-1 inhibitors are expected to be a new generation of anti-inflammatory drugs (22, 23). Our data revealed the perspectives of targeting mPGES-1 to attenuate PD-associated peritoneal fibrosis.

Previous studies have demonstrated that the expression of mPGES-1 is increased in inflammatory diseases, including colitis, arthritis, and gastritis (24–26). Our present study found that mPGES-1 is activated in the peritoneum of PD patients with UFF, and the expression was also increased in RPMCs treated with high glucose. The results from this study revealed that, in addition to the acute phase of diseases, mPGES-1 is still activated under sustained pathological stimulation, a finding that was consistent with the induction of mPGES-1 in peripheral blood mononuclear cells from hypertensive patients, kidney tissues of the unilateral ureteral obstruction mouse model and renal proximal tubular cells treated with albumin (27–29). Additionally, in our study, a positive correlation was found between the expression of mPGES-1 in the peritoneum and degree of peritoneal fibrosis. Thus, mPGES-1 maybe involved in the progression of peritoneal fibrosis. The results from our *in vitro* study further support that mPGES-1 plays an important role in the synthesis of fibrotic proteins in RPMCs, a pivotal event during peritoneal fibrosis. However, the role of mPGES-1 in fibrosis is controversial according to previous studies. In the unilateral ureteral obstruction mouse model, mPGES-1 exerts a potentially protective effect against renal fibrosis, while inhibition of mPGES-1 provides a viable method to alleviate the development of bleomycin-induced skin fibrogenesis (27, 30). These different effects on fibrosis may be attributed to different models of disease and tissue-specific mechanisms.

Peritoneal fibrosis during PD is a chronic inflammation-driven process, and PGE2 is a major proinflammatory mediator that is up-regulated during inflammation. Considering PGE2 is the terminal product of mPGES-1, we analyzed the relationship between the secretion of PGE2 in the dialysate and clinical characteristics of the PD patients. With a prolonged PD duration, the degree of peritoneal fibrosis became more severe. In our study, the expression of mPGES-1 increased with the aggravation of peritoneal fibrosis, and the secretion of PGE2 in the peritoneum of our PD patients was positively correlated with the PD duration. Additionally, PGE2 was negatively correlated with the residual urine volume and positively correlated with the serum creatinine level in our study. The major functions of the peritoneum during PD therapy include ultrafiltration of liquid and transport of small soluble molecules. A decreased residual urine volume and higher serum creatinine levels can represent worse peritoneal membrane function. Additionally, with a prolonged PD duration, the residual urine volume decreases, a finding that was also demonstrated in our study. Single isolated episodes of peritonitis have no significant effect on peritoneal creatinine transport (31); however, the results from the Global Fluid Study showed that intraperitoneal inflammation is the most important determinant of peritoneal small solute transport in incident PD patients (32). However, we found no statistical difference in the correlation between the intraperitoneal PGE2 levels and transport of small soluble molecules, partly due to the limitation of the enrollment number. More incident PD patients need to be recruited to further explore the relationship between the intraperitoneal PGE2 levels and small soluble molecule transport.

The results of our study showed a significant correlation between secretion of PGE2 and serum C-reactive protein. Serum C-reactive protein is a critical systemic biomarker of inflammation. A previous study indicated that during the first year of therapy, intraperitoneal and systemic inflammation increased in patients with PD treatment (33). Our results showed that, in PD patients, local intraperitoneal inflammation is associated with systemic inflammation. The results of a retrospective cohort study that recruited 402 PD patients demonstrated that the serum C-reactive protein level is an independent predictor of technique failure (34), suggesting that systemic inflammation is associated with technique survival.

mPGES-1/PGE2 plays a critical role in the progression of multiple diseases via proinflammatory mechanisms (28, 35, 36). We explored the effect between mPGES-1/PGE2 signaling and the NLRP3 inflammasome in high-glucose-stimulated RPMCs. Recently, increasing evidence has demonstrated the influence of mPGES-1/PGE2 on NLRP3 inflammation in different cell types. Blockade of COX-2/mPGES-1 could inhibit macrophage M1 polarization and NLRP3 inflammation activation in response to monosodium urate crystals (37). Additionally, PGE2 activates NLRP3 inflammation in endothelial cells to promote diabetic retinopathy (38). Furthermore, high-glucose-based PD solutions trigger NLRP3 inflammation activation in human peritoneal mesothelial cells (39), and angiotensin- (1-7) attenuated angiotensin-induced epithelial-mesenchymal transition in hepatocytes through inhibiting the NAPDH oxidase-derived, hydrogen peroxide-activated NLRP3 inflammation (40). In our study, we found that inhibiting NLRP3 in high-glucose-stimulated RPMCs attenuates the expression of extracellular matrix proteins.

Although this study demonstrated the positive relationship between mPGES-1/PGE2 and PD-related peritoneal fibrosis and the beneficial effect of the inhibition of mPGES-1 on the production of extracellular matrix proteins in RPMCs, the therapeutic effect targeting mPGES-1 were not evaluated in the PD animal model. Besides, mPGES-1/PGE2 exerts the functions through its downstream prostaglandin receptors. In this research work, we did not investigate the contribution of prostaglandin E2 receptors during the NLRP3 inflammasome activation and peritoneal fibrosis. Further studies are required to be performed to address these questions.

In summary, the present study demonstrated that mPGES-1/PGE2 signaling is activated during PD therapy, and the intraperitoneal PGE2 levels are associated with systemic inflammation and the PD duration. Furthermore, mPGES-1 contributes to increase the synthesis of extracellular matrix proteins of RPMCs by activating NLRP3 inflammation. Targeting mPGES-1 may attenuate the progression of peritoneal fibrosis during PD treatment.

## Data Availability Statement

The raw data supporting the conclusions of this article will be made available by the authors, without undue reservation.

## Ethics Statement

The studies involving human participants were reviewed and approved by Ethical Review Board of Shunde Hospital of Southern Medical University. The patients/participants provided their written informed consent to participate in this study.

## Author Contributions

ZJ and XD coordinated and oversaw the study. QL, QH, QZ, LG, LS, BR, and YJ collected samples, performed experiments, and analyzed data. QL, ZJ, and XD wrote the manuscript. All authors contributed to the article and approved the submitted version.

## Conflict of Interest

The authors declare that the research was conducted in the absence of any commercial or financial relationships that could be construed as a potential conflict of interest.
